# Ethyl 3-oxo-2-(2,5-dioxopyrrolidin-3-yl)butanoate Derivatives: Anthelmintic and Cytotoxic Potentials, Antimicrobial, and Docking Studies

**DOI:** 10.3389/fchem.2017.00119

**Published:** 2017-12-12

**Authors:** Fawad Mahmood, Muhammad S. Jan, Sajjad Ahmad, Umer Rashid, Muhammad Ayaz, Farhat Ullah, Fida Hussain, Ashfaq Ahmad, Arif-ullah Khan, Muhammad Aasim, Abdul Sadiq

**Affiliations:** ^1^Department of Pharmacy, Sarhad University of Science & Technology, Peshawar, Pakistan; ^2^Department of Pharmacy, University of Malakand, Chakdara, Pakistan; ^3^Department of Chemistry, COMSATS Institute of Information Technology, Abbottabad, Pakistan; ^4^Department of Pharmacy, University of Swabi, Swabi, Pakistan; ^5^Department of Pharmacology, Riphah Institute of Pharmaceutical Sciences, Riphah International University, Islamabad, Pakistan; ^6^Department of Biotechnology, University of Malakand, Chakdara, Pakistan

**Keywords:** succinimides, antibacterial, MICs, anthelminitic, *Ascaridia galli*, cytotoxicity, brine shrimps

## Abstract

Development of multidrug resistance (MDR) to antimicrobial, antiparasitic and chemotherapeutic agents is a global challenge for the scientific community. Despite of the emergence of MDR pathogens, the development of novel and more effective drugs is slow and scientist even speculate that we are going back the pre-antibiotic era. This work aims to study and evaluate the preliminary antibacterial, anthelmintic and cytotoxic potentials of ethyl 3-oxo-2-(2,5-dioxopyrrolidin-3-yl)butanoates. Among all of the four compounds, compound **2** has displayed remarkable potency with MIC values of 0.125, 0.083, 0.073, and 0.109 mg/ml against *E. sakazakii, E. coli. S. aureus*, and *K. pneumonia*, respectively. Compared to etoposide (LC_50_ 9.8 μg/ml), the compounds demonstrated LC_50_ values from 280 to 765 μg/ml. For anthelmintic assay, three concentrations of each compound and standard drug were studied in determination of time of death of the two species. Excellent anthelmintic activity was observed by all four compounds against *P. posthuma* and *A. galli* better than standard albendazole. High GOLD fitness score data from docking analysis toward the targets represent better protein–ligand binding affinity and thus indicate a high propensity for all the active compounds to bind to the active site. The promising *in-vitro* antimicrobial, anthelmintic activity, and cytotoxicity data conclusively revealed that these compounds may serve as viable lead compounds for the treatment of bacterial and parasitic infections, and therefore, could help the medicinal chemists to design future chemotherapeutic agents to avoid rapid drug resistance.

## Introduction

Microbial resistance and invading of infectious life-threatening diseases is a real challenge for scientists of the current era (Alanis, 2005; Ayaz et al., 2016a, 2017b). The prevalence of bacterial and parasitic infections is still a major risk in the developing countries (Okeke et al., 2005; Ullah et al., 2012). A major reason for the risk is the inappropriate, indiscriminate use of antimicrobials agents and unavailability of resistance modifying antibiotics against MDR pathogens (Wise et al., 1998; Ayaz et al., 2016a). The clinical efficacy of majority of antibiotics which were developed before 1970 has been endangered by the resistance pathogens (Livermore et al., 2011; Sadiq et al., 2016). To combat with the scenario of increasing over resistance of microorganisms, it is important to develop novel antibiotics preferably working with different mechanism of action (Kasanah and Hamann, 2004; Zeb et al., 2017). The results of susceptibility testing are very crucial for evaluation of microbial resistance and susceptibility (Reller et al., 2009). Susceptibility of a group of microbes varies to different chemical agents (Kümmerer, 2004). Till now, a vast research has been carried out in finding safe, effective, and economical antimicrobial drugs. The antimicrobial agents can be obtained from synthetic or natural sources (Herrlich and Schweiger, 1976; Ayaz et al., 2015b; Kamal et al., 2015a; Shah et al., 2015). The synthetic drugs are normally accompanied with unwanted side effects while the natural drugs possess broad spectrum of pharmacological activities and are more safe (Ayaz et al., 2014a, 2015a, 2017a; Ahmad et al., 2015, 2016b; Kamal et al., 2015b; Ali et al., 2016, 2017; Zeb et al., 2016). Therefore, there is a great need of new research for the development of new drugs which can be effective against several bacterial strains.

Helminthiasis, a common animal and human parasitic infection, is considered to be a next challenge for the developing scientists (Ayaz et al., 2014b). Approximately two billion people are suffering from parasitic diseases (Dhar et al., 1982). The helminthiasis can be accompanied with several complications like undernourishment, anemia, eosinophilia, and other physical and mental disorders (Blumenthal and Schultz, 1975). The parasitic infection in animals can cause a reduction in the milk (Olson et al., 2004). The helminthiasis is mostly associated to the poor hygiene and socio-economic problems (Tagboto and Townson, 2001). There is a low progress in the development of novel anthelmintic drugs which might be due to the financial output in comparison to the investment (Brown et al., 1961). Several anthelmintic agents have been designed and tested, but they are associated with certain limitations (Kamal et al., 2017). So, there is a dire need of the investigation of novel anthelmintic drugs.

Currently, neoplasia is a major cause of world's mortality (Östör, 1993). There are several causes of for the cytotoxicity (Valeriote and Van Putten, 1975). The clinicians are trying to eradicate this disease by several ways like radiotherapies and chemotherapies (Adams and Stratford, 1986). But due to the widespread risk of cancer, various anticancer agents have been demonstrated with overwhelming clinical outcomes (Hande, 1998). Anticancer agents available in the market include etoposide, vincristin, vinblastin, pacletaxel, docetaxel, and irinotecan (Haddadin and Perry, 2011). Due to the major risk of this disease for human life, various researchers are still trying to investigate new sources for the management of cancer (Zeb et al., 2014; Ahmad et al., 2016a).

Succinimides, also called 2,5-dioxopyrrolidine is an important class of drugs. Previously succinimides have been synthesized by a number of ways (Nugent et al., 2012; Chauhan et al., 2013; Sadiq et al., 2015). In the last few years, various derivatives of succinimides have been synthesized with efficient procedures (Nugent et al., 2012). The basic nucleus of succinimides can be derivative by both *C* and *N*-position leading to various derivatives with diverse functionalities. We have previously synthesized the ketoesters derivatives (ethyl 3-oxo-2-(2,5-dioxopyrrolidin-3-yl)butanoate) of succinimide with their promising anticholinesterase and antioxidant potential (Sadiq et al., 2015). In this specific article, we have re-synthesized the ketoesters derivatives of succinimides and have evaluated for antibacterial, anthelmintic, and cytotoxic assays.

## Materials and methods

### General information

All the reactions were set up in ordinary laboratory conditions in small vials of 2 ml. The liquid reagents were measured and transferred into the reaction vials with syringes. The solid samples were weighted using analytical balance and transferred to the reactions. Silica gel 60 F_254_ TLC plates were used and visualized under UV-lamp. The finely powdered silica gel (0.040–0.063 mm) was used for column chromatography with *n*-hexane and ethyl acetate as eluting solvents. NMR JEOL ECX 400 spectrophotometer, with proton NMR operating at 400 MHz was used. Chemical shifts (δ) of the signals were taken as parts per million downfield from the internal standard TMS. Multiplicities of lines were abbreviated as singlet (s), doublet (d), triplet (t), quartet (q), broad (br), and multiplet (m). The coupling constant values (*J*) were expressed in Hz.

### Synthesis of compounds

All the four compounds (**1–4**) were synthesized by conjugate additions of β-ketoesters to various *N*-phenyl or *N*-benzylmaleimides. The ketoesters(ethyl 2-oxocyclopentanecarboxylate or ethyl 2-oxocyclohexanecarboxylate) 2 mmol were added to the *N*-phenyl or *N*-benzylmaleimides (1 mmol). The reactions were catalyzed by combination of creatinine and KOH (each 20 mol%) in dichloromethane (1.0 M). The reaction was continued at room temperature and was monitored by thin layer chromatography. The disappearance of the limiting reagent (maleimides) was considered as completion point for each reaction. The stirring was stopped and the reaction mixture was added 15 ml of water and extracted with DCM three times (15 ml each). The combined organic (DCM) layers were dried with sodium sulfate (anhydrous). The mixture was filtered to remove Na_2_SO_4_ and filtrate was washed three times with DCM (3 × 15 ml).

### Purification of compounds

The combined organic layer containing the crude compound was concentrated and absorbed on silica gel (~2X g) surface with the help of rotary evaporator. The crude reaction was purified by column chromatography using *n*-hexane and ethyl acetate as eluting solvents. The final yield of each product was calculated from the obtained pure product in milligrams.

### Compound 1 [ethyl 2-oxo-1-(2,5-dioxo-1-phenylpyrrolidin-3-Yl)cyclopentanecarboxylate]

The purified product **1** appeared as yellowish semi-solid. The time of reaction was 20 h, isolated yield 95% and R_f_ value of 0.44 (in solvent system *n*-hexane/ethyl acetate, 4:1).

^1^H NMR (400 MHz, CDCl_3_) (ppm): 7.14–7.38 (m, 5H), 4.04–4.21 (m, 2H), 3.03–3.08 (m, 1H), 2.95 (dd, *J* = 9.5, 18.3 Hz, 1H), 2.41–2.71 (m, 2H), 2.35–2.41 (m, 3H), 1.71–2.24 (m, 2H), 1.15–1.21 (m, 3H).

### Compound 2 [ethyl 1-(1-benzyl-2,5-dioxopyrrolidin-3-Yl)-2-oxocyclopentanecarboxylate]

The purified compound **2** appeared as light brown viscous liquid. The reaction completed in 24 h with 91% isolated yield and R_f_ value of 0.54 (in solvent system *n*-hexane/ethyl acetate, 4:1).

^1^H NMR (400 MHz, CDCl_3_) (ppm): 7.09–7.40 (m, 5H), 4.04–4.15 (m, 4H), 3.09 (t, *J* = 9.1 Hz, 1H), 2.77–2.88 (m, 1H), 2.31–2.65 (m, 2H), 2.20–2.26 (m, 2H), 1.94–2.16 (m, 2H), 1.74–1.84 (m, 1H), 1.21 (t, *J* = 7.2 Hz, 3H).

### Compound 3 [ethyl 2-oxo-1-(2,5-dioxo-1-phenylpyrrolidin-3-Yl)cyclohexanecarboxylate]

The compound **3** was isolated with 98% yield in 22 h. The product appeared as viscous pale yellowish color liquid. The calculated R_f_ value of compound **3** was 0.55 in a solvent system *n*-hexane/ethyl acetate (4:1).

^1^H NMR (400 MHz, CDCl_3_) (ppm): 7.62 (dd, *J* = 3.5, 5.6 Hz, 1H), 7.45 (dd, *J* = 3.5, 5.5 Hz, 1H), 7.20–7.40 (m, 3H), 4.09–4.17 (m, 2H), 2.59–2.80 (m, 2H), 2.39–2.46 (m, 1H), 1.57–1.69 (m, 2H), 1.15–1.34 (m, 6H), 0.87 (t, *J* = 7.5 Hz, 3H).

### Compound 4 [ethyl 1-(1-benzyl-2,5-dioxopyrrolidin-3-yl)-2-oxocyclohexanecarboxylate]

Compound **4** was synthesized in 23 h with 84% isolated yield. The compound was brown oily with R_f_ value of 0.63 (*n*-hexane/ethyl acetate, 4:1).

^1^H NMR (400 MHz, CDCl_3_) (ppm): 7.18–7.33 (m, 5H), 4.54–4.65 (m, 2H), 4.09–4.18 (m, 2H), 3.14 (dd, *J* = 6.0, 9.2 Hz, 1H), 2.66–2.75 (m, 1H), 2.59 (dd, *J* = 9.2, 18.0 Hz, 1H), 2.39–2.45 (m, 1H), 1.75–1.80 (m, 1H), 1.58–1.70 (m, 2H), 1.20–1.36 (m, 4H), 0.86 (t, *J* = 7.5 Hz, 3H).

### *In Vitro* antibacterial activity

Four bacterial strains including *Enterobacter sakazakii, Escherichia coli, Staphylococcus aureus*, and *Klebsiella pneumonea* were kindly provided by Pharmacy Department, University of Malakand. Various biochemical tests were employed for its identification and were preserved at 4°C (Barrow and Feltham, 1993).

Bacterial strains were initially cultured on nutrient agar for 24 h at 37°C and suspensions corresponding to cell density of 1 × 10^8^ CFU ml^−1^ were prepared in nutrient broth using McFarland standards and were diluted to 1 × 10^6^ CFU ml^−1^ using a UV visible spectrophotometer (Thermo electron corporation, USA) at 625 nm. The standardization is very important in quantitative analysis and was sustained during the period of the study (Petrikkou et al., 2001).

The nutrient broth and agar methods were used to determine the MICs (Cruikshank et al., 1965; National Committee for Clinical Laboratory Standards, 1993; Ayaz et al., 2015c). For these tests, compounds 1–4 were prepared in concentration range 0.03–0.3 mg/ml and were added to sterilized tube containing nutrient broth. These were then inoculated with test microbes. The shaker incubator at 37°C for 24 h was used for incubation. The concentration at which no visible bacterial growth was observed was considered as MIC. All experiments were repeated three times.

### Anthelmintic assay

The adult earth worms (*P. posthuma*) and round worms (*A. galli*) were used in anthelmintic assay. The earth worms with an average length 7–8 cm and width 0.1–0.2 cm were collected from the damp soil. The round worms were collected from the intestine of freshly slaughtered domestic chickens weighing around 2 kg. Both types of worms were divided into different groups with six (6) worms in each group. Solutions for compounds (**1–4**) and albendazole were prepared in 5, 10, and 20 mg/ml concentrations each. The solution of each sample was transferred into sterilized Petri dish (150 × 15 mm). The groups of worms were transferred into the Petri dishes with sterilized forceps. The Petri dishes were labeled properly to avoid confusion and the times were monitored with stop watches. The paralysis and death times for each sample were recorded individually. The paralysis time was the time at which the worms lost their motilities while the death time was considered when the worms lost their motility, even with vigorous shaking in hot water at 50°C.

### Brine shrimps lethality assay

Brine shrimps (*Artemiasalina*) were used in determining the brine shrimps cytotoxicity assay (Ayaz et al., 2016b). A tray of dimension 22 × 32 cm with brine solution was taken for hatching the brine shrimps larvae. A partition was made in the tray with perforated plate. One half of the tray (containing 50 mg of *Artemiasalina* eggs) was covered with aluminum foil. The second half of the tray was a place for the newly hatched brine shrimps nauplii. The tray was kept at 37°C for 24 h to hatch the brine shrimps eggs. The newly hatched brine shrimps nauplii were attracted to the uncovered side of the tray and were collected with a Pasteur pipette. Thirty brine shrimps nauplii were added to each vial. The stock solution of each sample was prepared by dissolving 2 mg (of each compound separately) in 2 ml methanol. Three dilutions of 250, 500, and 1000 μg/ml concentrations of each sample were made in separate vials. The final volume of each vial was adjusted to 5 ml with sea water. Etoposide and sea water were used as positive and negative controls respectively. The vials were kept at 25°C for 24 h. At 24 h, the numbers of alive and dead brine shrimps nauplii were observed with the help of a magnifying glass. All the experiments were performed in triplicate.

Data is represented as mean ± SEM *n* = 3. Two-way ANOVA followed by Bonferroni test was applied for significant difference between positive control and test compounds at 95% confidence interval. The values were significantly different with ^*^P < 0.05, ^**^P < 0.01, and *P* < 0.001.

### Docking studies

Docking experiment was carried out using GOLD docking program 5.4.1 (Verdonk et al., 2003). GOLD uses the Genetic algorithm (GA). This method allows a partial flexibility of protein and full flexibility of ligand. The X-ray crystallographic structure of PBP 2A from *S. aureus* (PDB ID 1VQQ) and β-lactamase NDM-1 from *Klebsiella pneumonia* (PDB ID 3Q6X) was used as a protein structure. In the preliminary preparation of pdb files, the chain selection, multiple ligands, or non-protein parts, deletion of water molecules were performed using UCSF chimera package. Energy minimization of downloaded proteins were carried out using default parameters of Chimera 1.11.2rc (steepest descent minimization algorithms = 100, step size = 0.02 Å and update interval = 10; Pettersen et al., 2004). In case of 3Q6X, active site of the prepared protein was defined as the residues within 10 Å of the reference ligand (ampicillin). In case of protein with no co-crystalized ligand (1 VQQ), the allosteric site was selected and defined from the list of amino acid residues reported in the literature (Kumar et al., 2014; Rani et al., 2016). The important residues are: Asn146, Ser148, Ser149, Lys148, Lys273, Val277, Gln292, His293, Glu294, Asp295, Tyr297, Arg298, Val299, Thr300, Ile314, Glu315, and Lys316).As a first step, the ability of the docking algorithm was validated to reproduce the co-crystallized pose of ampicillin in the 3Q6X pocket. The validation was carried out using RMSD and cross-docking method. In case of 3Q6X, the co-crystalized ligand was re-docked and the root mean square deviation (RMSD) between co-crystallized (ampicillin) and re-docked conformation was determined. The RMSD value of < 2.0 Å is considered as accurate in predicting binding orientation of ligand (Cole et al., 2005). For 1VQQ, a protein model was built by performing docking on ceftriaxone into the binding site. This model was then subjected to energy minimization and further used for docking studies of the synthesized compounds. There were four possible scoring functions: Goldscore, Chemscore, Astex Statistical Potential (ASP), and ChemPLP. GoldScore performs a force field based scoring function and is made up of four components: 1. Protein-ligand hydrogen bond energy (external H-bond); 2. Protein-ligand van der Waals energy (external vdw); 3. Ligand internal van der Waals energy (internal vdw); 4. Ligand intramolecular hydrogen bond energy (internal-H-bond). Binding site was defined as the residues within 6 Å from the ligand. No water was present in any binding site. The default docking protocol was applied (1.09 auto settings, 10 GA) and the best pose saved. Each experiment was then repeated 10 times. Other docking parameters were set to the software's default values. The view of the docking results and analysis of their surface with graphical representations were done using Discovery Studio Visualizer and UCSF Chimera package (Pettersen et al., 2004).

## Results and discussion

### Chemistry

**Figure 1:**
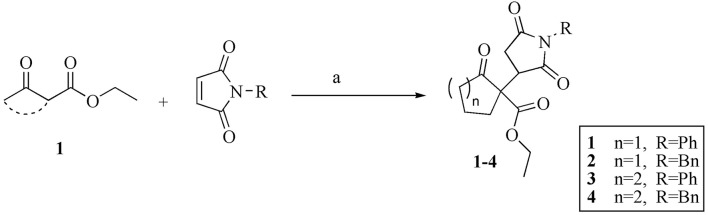
Creatinine promoted Michael addition of β-ketoesters to maleimides. Reaction conditions: (a) Creatinine (20 mol%), KOH (20 mol%), DCM (1.0 M), rt, 20–24 h.

We synthesized the compounds **1–4** by creatinine promoted conjugate addition of β-ketoesters (**1**) to substituted male imides (Figure 1). All the four compounds (**1–4**) were obtained in relatively shorter time of reaction and with excellent isolated yields. The isolated yields for compounds 1, 2, 3, and 4 were 95, 91, 98, and 84%, respectively. The compounds were identified by comparing the R_f_ values and ^1^H NMR data with our previous report on the same types of compounds (Sadiq et al., 2015).

### *In Vitro* antibacterial activity

Succinimides is an important biologically active class of molecules (Patil and Rajput, 2014). Besides their medicinal importance, they are also considered as building blocks for other important drugs. The pyrrolidine-2,5-dione ring can be reduced or changed into lactams, which are important drug molecules (Xiao et al., 2010). Generally, succinimides are known for the treatment of epilepsy. The commonly employed succinimides are phensuximide, mesuximide, and ethosuccimide. Besides their anti-convulsant properties, various derivatives of succinimides have broad range of pharmacological activities (Patil and Rajput, 2014). The succinimide ring (pyrrolidine-2,5-dione) can be synthesized in a number of ways. Currently, the most practical approach is to add a nucleophilic agent at the beta position of an α,β-unsaturated maleimide. This approach can lead to a diverse array of both *N*-and *C*-substituted succinimide derivatives. Based on the diverse range of possible derivatives for the basic nucleus, the succinimides can have verities of pharmacological potentials.

**Figure 2:**
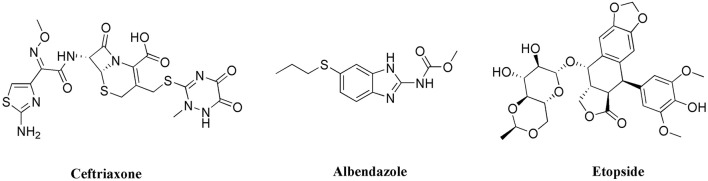
Structures of standard drugs used in comparison to succinimides.

To establish a common comparison between the synthesized succinimides and the standard drugs used in the study, we have shown the structures of these compounds in Figure 2. The succinimide is basically a five member ring with fused nitrogen atom and two carbonyl groups. In this case, the basic nucleus of succinimide is extended with an aromatic ring at the *N*-position and ketoester moiety at *C*-position. So overall, the synthesized succinimides contain an aromatic ring, amide moiety, a cyclic ketone and an ester group. Though, there is no aromatic ring in the chemical structure of ceftriaxone, but it contains different amide moieties, carbonyl groups, and heterocyclic rings. So, the higher antibacterial activity of the tested succinimides might be attributed to the functional groups similarities with the standard drug. Moreover, the albendazole molecule also contains amide group, heterocyclic functionality and an aromatic ring. So, greater functional groups/structural similarities might have an influence on it anthelmintic activity as can be seen in Tables 1, 2. Alternatively, if we look to the structure of etoposide which has lots of cyclic system and mostly there are oxygen atoms in the molecules. The functional groups comparison between the succinimide derivatives and etoposide is less likely matching which might be a possible reason for lower cytotoxicity as shown in the Table 3.

**Table 1:** Anthelmintic activity of the synthesized ethyl 3-oxo-2-(2,5-dioxopyrrolidin-3-yl)butanoate derivatives against *Pheretima posthuma*.

**Compound**	**Conc. (mg/ml)**	**Paralysis time (min)**	**Death time (min)**
1	5	10.50 ± 2.29^ns^	41.33 ± 1.52^ns^
	10	7.33 ± 2.56^ns^	20.66 ± 1.15^**^
	20	4.66 ± 1.52^ns^	11.33 ± 1.52^***^
2	5	10.33 ± 1.15^ns^	34.66 ± 0.57^**^
	10	9.66 ± 2.51^ns^	24.33 ± 1.15^*^
	20	8.66 ± 2.30^ns^	21.66 ± 2.30^ns^
3	5	14.33 ± 0.57^ns^	23.83 ± 1.04^***^
	10	6.66 ± 0.57^ns^	10.90 ± 0.85^***^
	20	5.66 ± 1.15^ns^	8.40 ± 0.36^***^
4	5	10.33 ± 0.57^ns^	18.30 ± 0.36^***^
	10	8.66 ± 1.15^ns^	12.43 ± 0.40^***^
	20	3.66 ± 1.15^ns^	7.60 ± 0.17^***^
Albendazole	5	12.66 ± 1.52	49.67 ± 1.52
	10	10.00 ± 1.00	37.67 ± 0.57
	20	7.67 ± 0.57	29.33 ± 1.54

*Data is represented as mean ± SD, n = 3. Values significantly different in comparison to albendazole treated group*.

* *P < 0.05*,

** P < 0.01, and

*** *P < 0.001. ns, Values not significantly different in comparison to standard drug treated group*.

**Table 2:** Anthelmintic activity of the synthesized ethyl 3-oxo-2-(2,5-dioxopyrrolidin-3-yl)butanoate derivatives against *Ascaridia galli*.

**Compound**	**Conc. (mg/ml)**	**Paralysis time (min)**	**Death time (min)**
1	5	10.66 ± 2.52^ns^	64.16 ± 2.75^*^
	10	8.44 ± 1.50^ns^	48.33 ± 1.52^ns^
	20	6.83 ± 1.04^ns^	41.86 ± 3.09^*^
2	5	3.73 ± 1.16^*^	43.23 ± 3.52^ns^
	10	3.23 ± 1.05^*^	35.83 ± 1.25^ns^
	20	2.16 ± 1.02^***^	34.33 ± 2.02^ns^
3	5	3.93 ± 1.61^**^	41.90 ± 2.11^ns^
	10	1.73 ± 0.51^***^	38.50 ± 1.32^ns^
	20	1.97 ± 1.32^***^	35.20 ± 2.52^ns^
4	5	3.23 ± 1.16^***^	26.00 ± 2.78^***^
	10	1.87 ± 1.002^***^	21.50 ± 1.32^***^
	20	1.69 ± 1.04^***^	18.66 ± 2.56^***^
Abendazole	5	13.1 ± 1.85	47.40 ± 1.50
	10	9.80 ± 1.60	40.20 ± 2.25
	20	7.10 ± 1.01	33.60 ± 1.76

*Data is represented as mean ± SD, n = 3. Values significantly different in comparison to albendazole treated group*.

* *P < 0.05*,

** P < 0.01, and

*** *P < 0.001. ns, Values not significantly different in comparison to standard drug treated group*.

**Table 3:** Cytotoxic potential of the synthesized ethyl 3-oxo-2-(2,5-dioxopyrrolidin-3-yl)butanoate derivatives against Brine shrimps nauplii.

**Compound**	**Brine shrimps treated**	**Conc. (μg/ml)**	**Percent cytotoxicity (mean ± *SD*)**	**LC_50_ (μg/ml)**
1	30	1,000	73.33 ± 0.57^ns^	325
		500	58.33 ± 1.52^*^	
		250	46.66 ± 1.52^ns^	
2	30	1,000	62.33 ± 1.52^***^	765
		500	37.33 ± 0.57^***^	
		250	23.66 ± 0.57^***^	
3	30	1,000	67.66 ± 1.15^**^	395
		500	55.66 ± 2.08^ns^	
		250	41.33 ± 2.30^*^	
4	30	1,000	76.66 ± 1.15^ns^	280
		500	57.33 ± 1.52^ns^	
		250	49.33 ± 2.08^ns^	

*Values are expressed as the mean ± SEM of three independent observations. Standard drug; Etoposide LC_50_ = 9.8 μg/ml. Values significantly different in comparison to etoposide treated group*.

* *P < 0.05*,

** P < 0.01, and

*** *P < 0.001. ns, Values not significantly different in comparison to standard drug treated group*.

**Figure 3:**
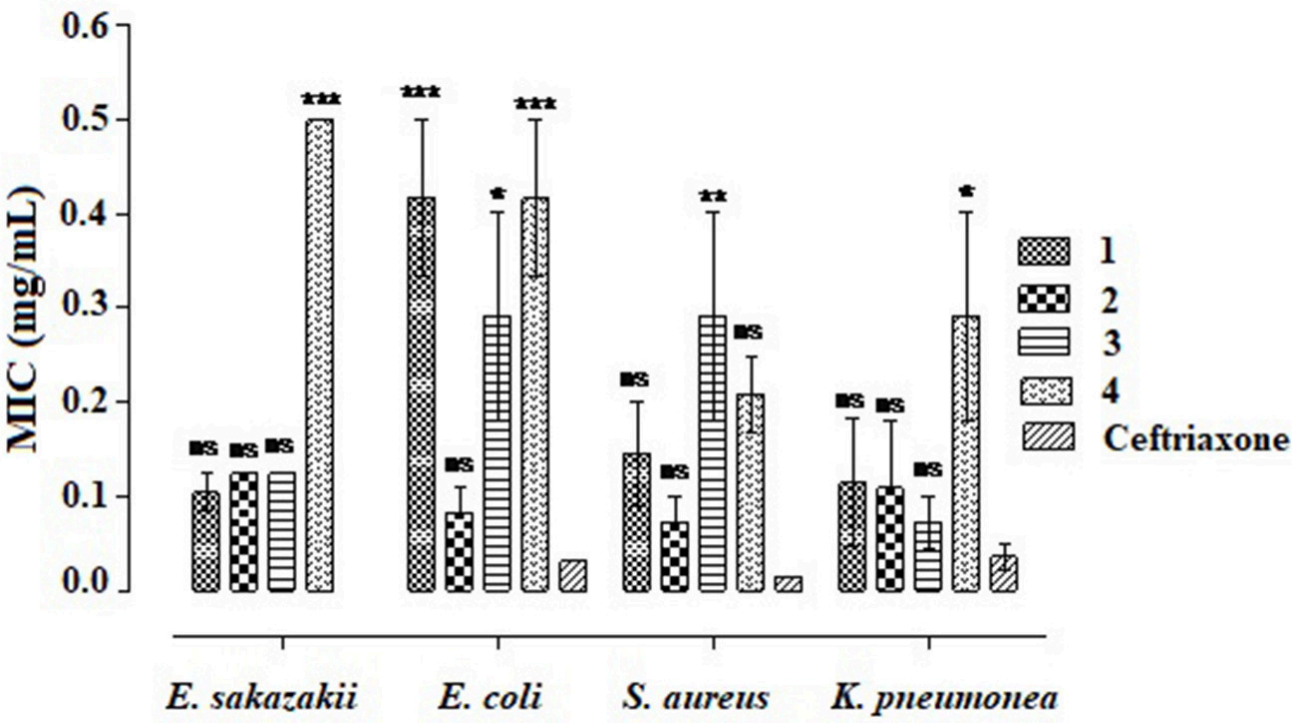
Determination of minimum inhibitory concentrations of ethyl 3-oxo-2-(2,5-dioxopyrrolidin-3-yl)butanoate derivatives. Values significantly different in comparison to the standard drug. ^*^P < 0.05, ^**^P < 0.01, and ^***^P < 0.001. ns, Values not significantly different in comparison to standard drug treated group.

The minimum inhibitory concentrations (MICs) of the four compounds (**1–4**) were determined using bacterial strains like *E. sakazakii, E. coli, S. aureus*, and *K. pneumonia* as shown in Figure 2. Overall, compound **2** was dominant against all the bacterial strains exhibiting MIC values of 0.13 ± 0.00, 0.08 ± 0.03, 0.07 ± 0.02, and 0.11 ± 0.07 mg/ml against *E. sakazakii, E. coli, S. aureus*, and *K. pneumonia*, respectively. In comparison, the standard drug ceftriaxone reveal 0.00, 0.031 ± 0.00, 0.015 ± 0.00, and 0.036 ± 0.013 mg/ml MICs against *E. sakazakii, E. coli, S. aureus*, and *K. pneumonia*, respectively. Among all, **compound4** was least effective against all the tested strains as obvious from Figure 3. Moreover, compounds **1**, **2**, and **3** were comparatively effective against *E. sakazakii* giving 0.10 ± 0.02, 0.13 ± 0.00, and 0.13 ± 0.00 mg/ml MICs, respectively. Similarly, these three compounds were also effective against *K. pneumonia* exhibiting MIC values of 0.11 ± 0.07 (**1**), 0.11 ± 0.07 (**2**), and 0.07 ± 0.03 (**3**) mg/ml.

### Anthelmintic activity

The anthelmintic assay was performed using earthworms (*P. pothuma*) and roundworms (*A. galli*) with albendazole as standard drug as shown in Tables 1, 2, respectively. The earth worms were used due to their close physiologic and anatomic similarity with that of human intestinal worms *Ascarislum bricoides* (Ayaz et al., 2014b). However, scientific evidence has shown that mostly *A. galli* are used to determine the anthelmintic assay due to its high resemblance with *A. bricoides*. *A. galli* are found in the intestine of domestic chickens having weight more than 1 kg.

In *P. posthuma* lethality assay, all the compounds showed overwhelming paralysis and death times at all the three concentrations excelling the standard drug albendazole as shown in Table 1. Among all the compounds, compound **4** was much excellent in showing the shortest death times at all tested concentrations. Compound **4** exhibited 18.30 ± 0.36, 12.43 ± 0.40, and 7.60 ± 0.17 min death times at 5, 10, and 20 mg/ml concentrations, respectively. In comparison, the standard drug albendazole was observed with longer death times, i.e., 49.67 ± 1.52, 37.67 ± 0.57, and 29.33 ± 1.54 min at 5, 10, and 20 mg/ml, respectively. Similarly, compounds **1**, **2**, and **3** demonstrated 11.33 ± 1.52, 21.66 ± 2.30, and 8.40 ± 0.36 min deaths time at 20 mg/ml.

The results of anthelmintic assay using *A. galli* are summarized in Table 2. Likewise *P. posthuma* assay, all the compounds showed excellent anthelmintic potential with compound 4 the leading one. Again, compound **4** exhibited 26.00 ± 2.78, 21.50 ± 1.32, and 18.66 ± 2.56 min death times at 5, 10, and 20 mg/ml, respectively. In comparison, the standard drug albendazole was observed with 47.40 ± 1.50, 40.20 ± 2.25, and 33.60 ± 1.76 min deaths times at 5, 10, and 20 mg/ml concentrations respectively. Similarly, compounds **1**, **2**, and **3** also revealed shorter deaths times of 41.86 ± 3.09, 34.33 ± 2.02, and 35.20 ± 2.52 min, respectively at 20 mg/ml.

### Brine shrimps cytotoxicity

In brine shrimps cytotoxicity assay, all the compounds (**1–4**) showed mediocre results in the lethality of brine shrimps nauplii as shown in Table 3. Among the tested compounds, **4** and **1** were relatively better with 76.66 ± 1.15 and 73.33 ± 0.57% lethality, respectively at 1,000 μg/ml. The calculated LC_50_ values were 325, 765, 395, and 280 μg/ml for compounds **1, 2, 3**, and **4**, respectively. The LC_50_ for etoposide was 9.8 μg/ml.

### Docking studies

The aim of docking studies is to predict the binding poses of the synthesized compounds. This is also referred to as docking accuracy or reliability. We evaluated the docking accuracy as root mean square deviation (RMSD) between the experimentally determined position (i.e., of co-crystallized ligand) and docked position. In case of 3Q6X, the co-crystalized ligand was re-docked and the root mean square deviation (RMSD) between co-crystallized (ampicillin) and re-docked conformation determined was 0.89 Å. The RMSD value of < 2.0 Å is considered as accurate in predicting binding orientation of ligand (Cole et al., 2005). There are many other methods that are used for the docking validations. One of them is leave-one-out (LOO) method (Yadav et al., 2014a,b). But all these methods are used for big data bases in Quantitative Structure Activity Relationship (QSAR) or Virtual Screening Experiments. However, in current study we apply a short cross docking experiments on multiple protein structures from protein data bank (PDB). The PDB codes of these structure and their co-crystallized ligands are shown in Table S1. We extracted all the four NDM-1 ligands from the given PDBs and are used to evaluate the docking poses. A combination of native and non-native docking experiments confirmed the validity of our docking accuracy. The results of the cross docking experiment are shown in Figure S1.

Interactions of the synthesized compounds with important residues were determined by docking studies. Computational docking studies were carried out using GOLD (Genetic Optimization for Ligand Docking) suit *v*5.4.1 to understand the types of interactions and binding orientations. While using bacterial strains, it is difficult to hypothesize the biomolecular antibacterial target that could help us to explore the mechanism. In order to provide some insight, we were in opinion that standard drug used in this study are of importance to study the mechanism. Ceftriaxone is a third generation from cephalosporin family and is used to treat organisms that are resistant to other antibiotics. It is highly potent against Gram positive and Gram negative bacteria. Ceftriaxone inhibit the final step in the cell wall biosynthesis by binding to penicillin binding proteins (PBPs). PBPs are membrane-associated enzyme and have been the subject of much research as the target of β-lactam antibiotics. Bacteria possess a large number of PBPs found as both membrane bound and cytoplasmic proteins (Goffin and Ghuysen, 1998; Macheboeuf et al., 2006; Sauvage et al., 2008). The antibacterial activity of β-lactam antibiotics depends on the binding affinity to specific PBPs and high affinity toward any one of the PBPs is considered to be enough for the antibacterial activity.

**Figure 4:**
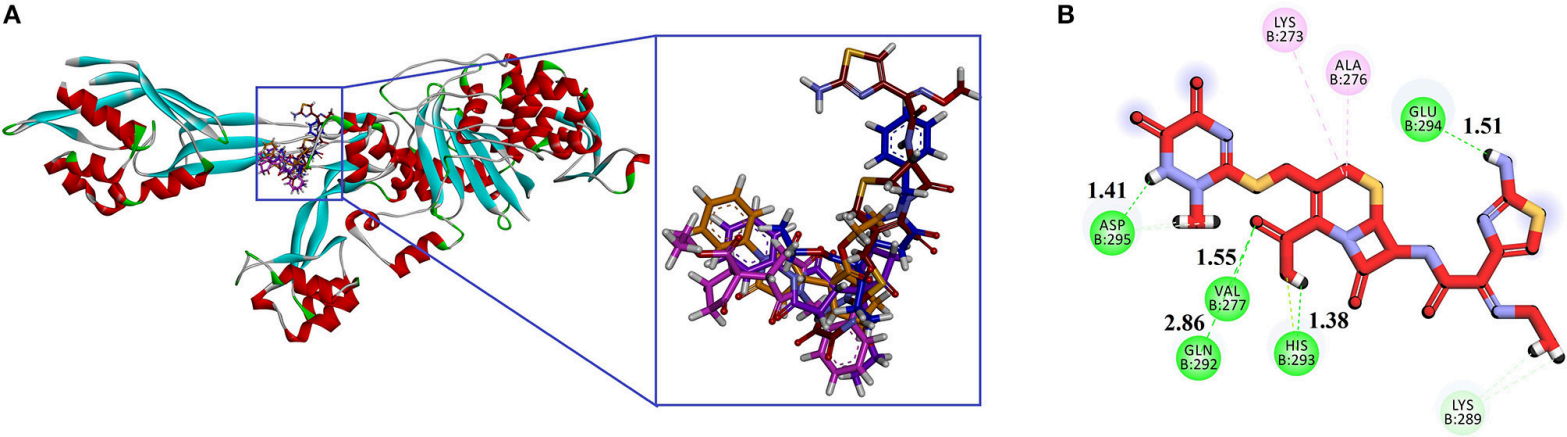
**(A)** Superimposed binding modes of compounds **1** (purple), **2** (pink), **3** (light yellow), **4** (blue) and Ceftriaxone (red) in the active site of PBP 2A from *S. aureus* (PDB ID 1VQQ); **(B)** 2D interaction plot of ceftriaxone generated by Discovery Studio Visualizers showing interaction of with key amino acid residues. Conventional hydrogen bonding is shown by green color dotted lines.

We selected crystal structure of PBP 2A (from *S. aureus*) retrieved from PDB (PDB ID 1VQQ) for docking studies. The important amino acid residues in the binding site are: Asn146, Ser148, Ser149, Lys148, Lys273, Val277, Gln292, His293, Glu294, Asp295, Tyr297 (Kumar et al., 2014). Goldscore was selected as the criteria for the selection of compounds because it serves as fitness function for the orientation, and estimates of binding affinity. Superimposed binding modes of all four ethyl 3-oxo-2-(2,5-dioxopyrrolidin-3-yl)butanoate derivatives and standard drug ceftriaxone in the active site of 1VQQ are shown in Figures 4A,B. Ceftriaxone exhibited GOLD fitness score (75.37) with five conventional hydrogen bonds (HBs) with Val277, Gln292, His293, Glu294, and Asp295 (Figure 4B). This high Gold Score represents its better binding affinity.

**Figure 5:**
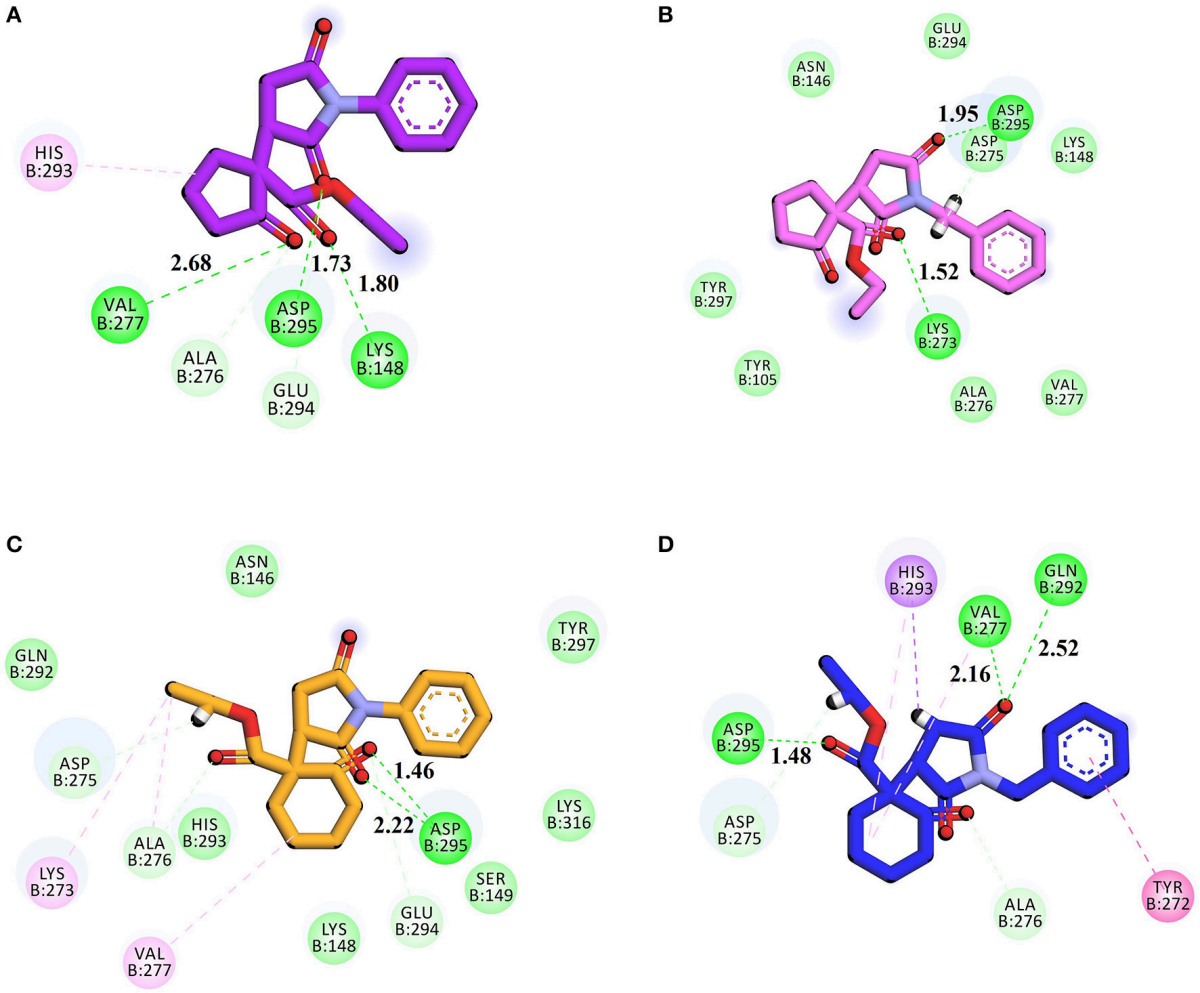
**(A-D)** 2D interaction plot of Compound **1–4** generated by Discovery Studio Visualizer showing interaction of with key amino acid residues. Conventional hydrogen bonding is shown by green color dotted lines.

Gold fitness score for compound **1** is 67.39 which may indicate that it has strong affinity with PBP. It forms three HBs with Lys148 (1.80 Å), Val277 (2.68 Å), and Asp295 (1.73 Å; Figure 5A). Compound **2** forms HB interactions with two amino acids i.e. Lys273 (1.53 Å) and Asp295 (1.95 Å; Figure 5B). Similarly, compounds **3** and **4** form two and three HBs, respectively. The 2D interactions of compounds **3** and **4** are shown in Figures 5C,D, respectively.

**Figure 6:**
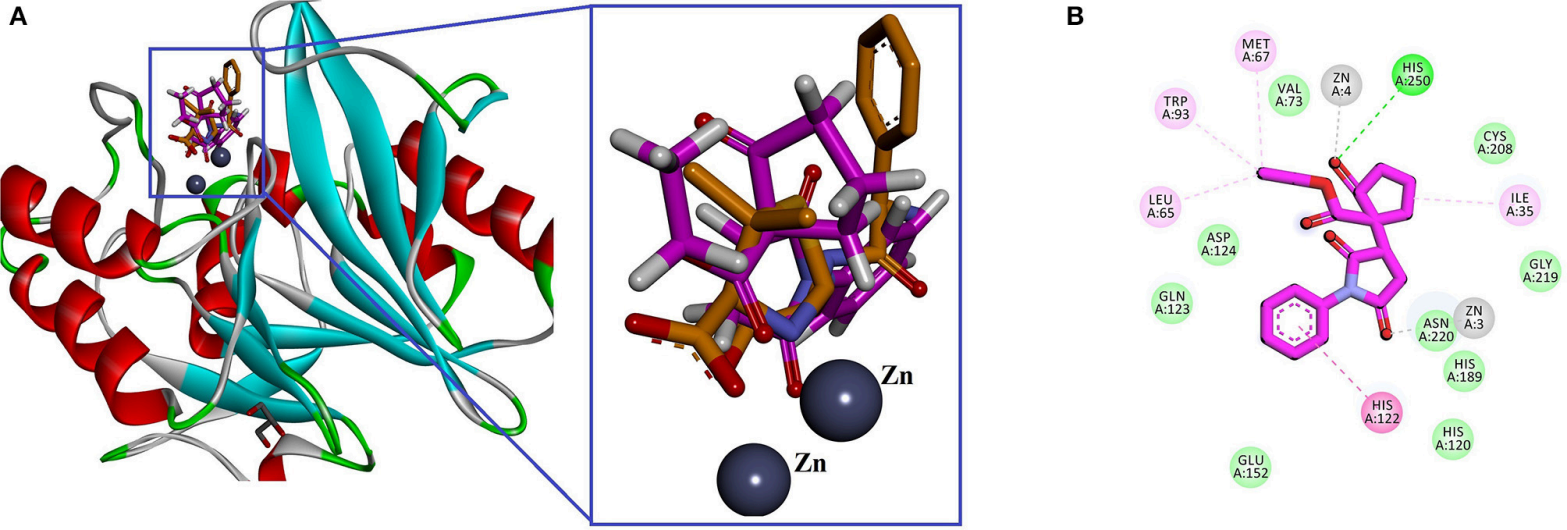
**(A)** Superimposedlowest energy binding pose of compound **1** **(**pink**)** with ampicillin (brown), the binding site of β-lactamase NDM-1 from *Klebsiella pneumonia* (PDB ID 3Q6X). Zinc atoms are shown in spheres; **(B)** 2D interaction plot of compound **1** generated by Discovery Studio Visualizer.

In search of antibacterial mechanism of the ketoester derivatives of succinimides, we extended our study and docking analysis was carried out on β-lactamase. Cephalosporin antibiotics contain β-lactam nucleus in their structure and inhibit the peptidoglycan layer of cell wall. However, bacteria resist β-lactam antibiotics due to the production of β-lactamases that catalyzes the hydrolysis of these antibiotics and rendering the antibiotics inactive. Based on amino acid sequences, β-lactamases are classified into four classes (A–D) and among them class B are metalloβ-lactamase (MBL). MBLs contain two divalent Zn as co-factors in the active site (Bush et al., 1995; Bush, 2010; Bush and Jacoby, 2010). New Delhi Metallo-Beta-Lactamase (NDM-1), named after its city of origin, belongs to β-lactamases. It was identified in a patient with a resistant *K. pneumonia* infection (Yong et al., 2009). Here, we hypothesized that our synthesized compounds may have some potential effect to inhibit NDM-1. We selected crystal structure of β-lactamase NDM-1 from *K. pneumonia* in complex with ampicillin was retrieved from PDB (PDB ID 3Q6X) for docking studies. The amino acid residues present in the active site are: Leu65, His120, His122, Gln123, Asp124, His189, Cys208, Lys211, Asn220, Ser249, His250, and two Zn ions (Zhang and Hao, 2011; Chiou et al., 2014). Almost all the four compounds occupy the same place as that of ampicillin (co-crystallized ligand; Figure 6A). The 2D interaction of compound **1** is shown in Figure 6B. His250 establishes a conventional hydrogen bond with carbonyl oxygen of cyclopentanone. One of the Zn ion is also coordinated with the His250. While, the second Zn ion coordinated with the carbonyl oxygen of Succinimide. His122 forms a π-π stacking interaction with phenyl ring. The calculated GOLD fitness score lies in range of 70.27–68.82.

## Conclusion

In closing, we have shown that ketoester derivatives of Succinimides can be used as antibacterial, anthelmintic and cytotoxic agents. Compound **2** was dominant in antibacterial assay. All the compounds demonstrated mediocre cytotoxic potential with compound **4** comparatively more effective. All the compounds were excellent in anthelmintic assay against both *P. posthuma* and *A. galli*. The comparative death times shown by compound **4** were more potent than the standard drug. In order to study the possible mechanism of antibacterial activity, computational docking studies were carried out using GOLD docking suite. Two target proteins namely Penicillin binding protein (PBP) and β-lactamase NDM-1 from *K. pneumonia* were studied. The binding mode analysis of the synthesized compounds suggests that these two proteins may be the target for their antibacterial activity.

## Author contributions

FM, SA, FH, and MJ: Synthesized and purified the compounds; MJ, SA, MAa, and AA: Performed the biological studies; FU and AK: Provided the chemicals for all biological assays and helped in relevant literature; MAy: Performed the statistical analysis and refined the manuscript; UR: Performed the docking studies; AS: Supervised the overall research work, drafted the manuscript, and refined for publication.

### Conflict of interest statement

The authors declare that the research was conducted in the absence of any commercial or financial relationships that could be construed as a potential conflict of interest.

## Abbreviations

*E. sakazakii*: Enterobacter sakazakii
*E. coli*: Escherichia coli
*S. aureus*,: Staphylococcus aureus
*K. pneumonia*: Klebsiella pneumonea
*P. posthuma*: Pherethima posthuma
*A. galli*,: *Ascaridi agalli;*
LC_50_: median lethal dose
MIC: minimum inhibitory concentration
TLC: thin layer chromatography
ppm: parts per million
δ: delta (represent chemical shift)
CDCl_3_: deutrated chloroform
R_f_: Retardation factor.
