# Supplementary material for: Ethyl 3-oxo-2-(2,5-dioxopyrrolidin-3-yl)butanoate Derivatives: Anthelmintic and Cytotoxic Potentials, Antimicrobial, and Docking Studies
Source: Front Chem. 2017 Dec 12;5:119. doi: 10.3389/fchem.2017.00119 (PMC5733081; doi:10.3389/fchem.2017.00119)

**Ethyl 3-oxo-2-(2,5-dioxopyrrolidin-3-yl)butanoate derivatives: Anthelmintic and cytotoxic potentials, antimicrobial and docking studies**

Fawad Mahmood^1^, ([fawadpharmacist@gmail.com](mailto:sadiquom@yahoo.com))

Muhammad Saeed Jan^2^, ([saeedjanpharmacist@gmail.com](mailto:arifullahkhan979@hotmail.com))

Sajjad Ahmad^2^, ([drsajjaduom@gmail.com](mailto:arifullahkhan979@hotmail.com))

Umer Rashid^3^, ([umerrashid@ciit.net.pk](mailto:umerrashid@ciit.net.pk))

Muhammad Ayaz^2^, ([ayazuop@gmail.com](mailto:ayazuop@gmail.com))

Farhat Ullah^2^, ([farhataziz80@hotmail.com](mailto:farhataziz80@hotmail.com))

Fida Hussain^2,4^, ([fida2k9@yahoo.com](mailto:fida2k9@yahoo.com))

AshfaqAhmad^1^([ashfaq_pharma@yahoo.com](mailto:zeb.takor@yahoo.com))

Arif-ullah Khan^5^ ([arif.ullah@riphah.edu.pk](mailto:arif.ullah@riphah.edu.pk))

Muhammad Aasim^6^ ([takkar4u@yahoo.com](mailto:takkar4u@yahoo.com))

Abdul Sadiq^*2^([sadiquom@yahoo.com](mailto:sadiquom@yahoo.com))

**Running title:** Synthesis and biological evaluation of Ethyl 3-oxo-2-(2,5-dioxopyrrolidin-3-yl)butanoate derivatives

^1^Department of Pharmacy, Sarhad University of Science & Technology, Peshawar, KPK, Pakistan

^2^Department of Pharmacy, University of Malakand, Chakdara, 18000 Dir (L), KPK, Pakistan

^3^Department of Chemistry, COMSATS Institute of Information Technology, Abbottabad 22060, Pakistan

^4^Department of Pharmacy, University of Swabi, Swabi, KPK, Pakistan

^5^Department of Pharmacology, Riphah Institute of Pharmaceutical Sciences, Riphah International University, Islamabad, Pakistan

^6^Department of Biotechnology, University of Malakand, Chakdara, 18000 Dir (L), KPK, Pakistan

*Assistant Professor, Department of Pharmacy, University of Malakand, Chakdara, 18000 Dir (L), KPK, Pakistan,Email: [sadiquom@yahoo.com](mailto:sadiquom@yahoo.com), Contact: +92 (0)301-2297 102

**Supplementary Information**

**Table S-1:** Crystal structures information New Delhi Metallo-Beta-Lactamase (NDM-1) downloaded from PDB

| PDB code | Co-crystalized ligand code | Resolution  (Å) | Organism |
| --- | --- | --- | --- |
| 3Q6X (Native) | ZZ7 (Native) | 1.3 | *Klebsiella pneumonia* |
| 4EXS | X8Z | 2.4 |  |
| 4EY2 | 0RM | 1.17 |  |
| 4EYB | 0WO | 1.16 |  |


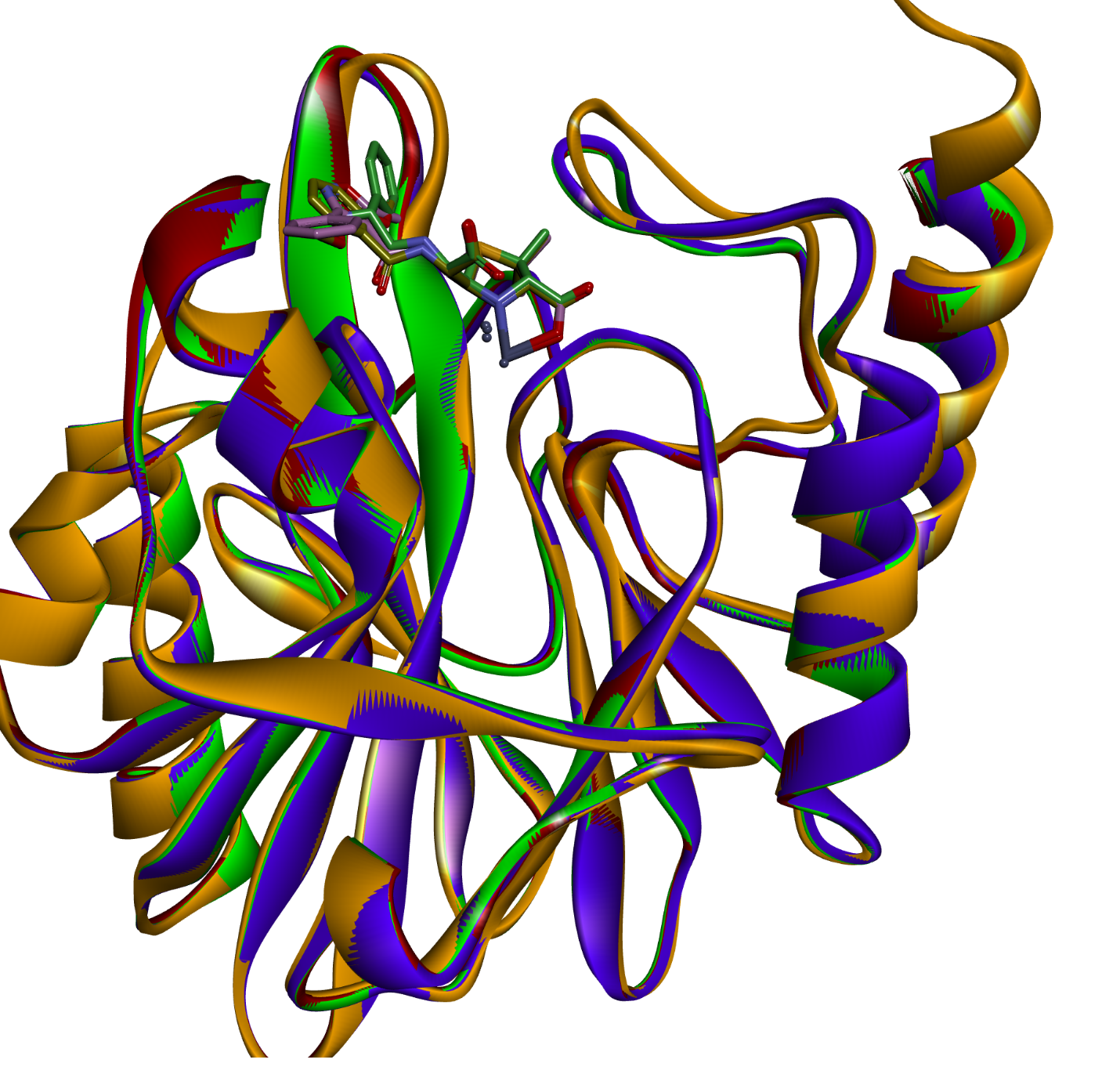


**Figure S-1:**A combination of native and non-native cross docking experiments confirmed the validity of our docking accuracy. Superimposition of some of docked non-native ligands and protein structures on native ligand (**ZZ7**) of 3Q6X for NDM-1. The superimposition shows that all the non-native ligands are docked on the same position of native ligand. Blue ribbons shows 3Q6X and its native ligand, red for 4EXS, light green for 4EY2 and light brown for 4EYB.

**^1^H NMR spectrum of compound 1**
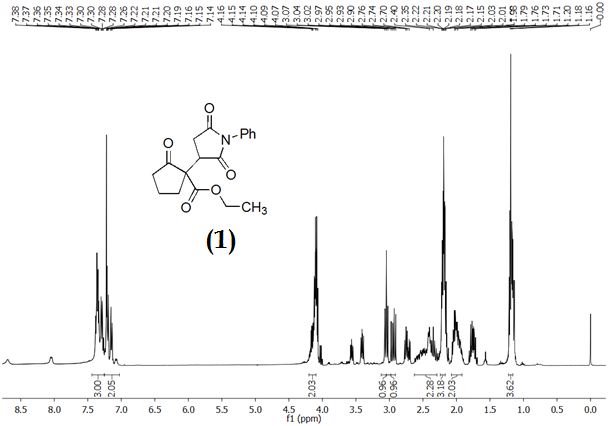


**^13^C NMR of compound 1**


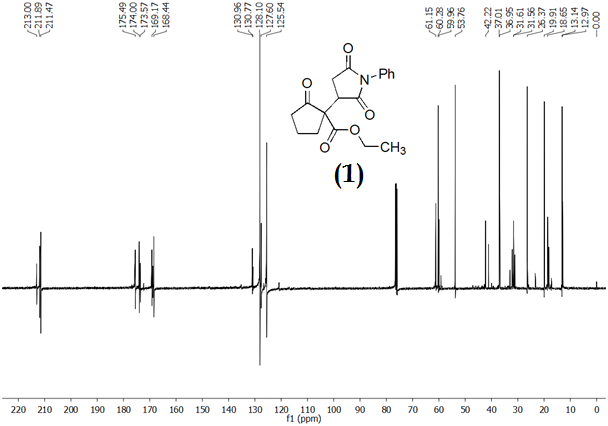


**^1^ H NMR spectrum of compound 2**


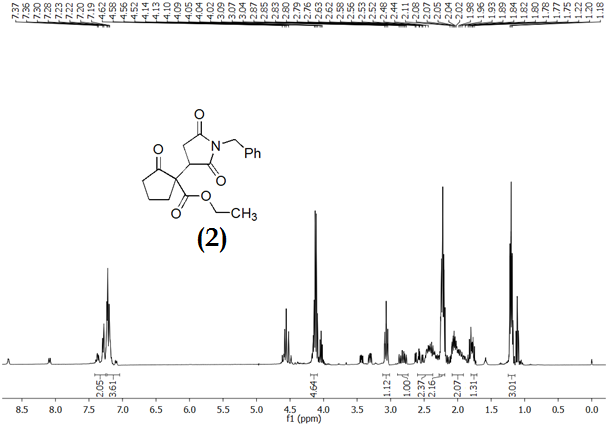


**^13^C NMR spectrum of compound 2**


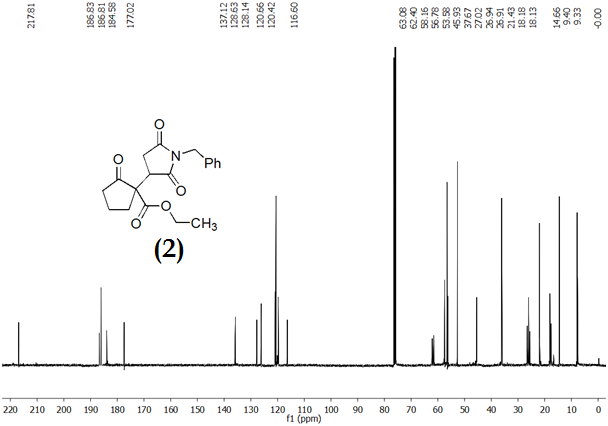


**^1^H NMR spectrum of compound 3**


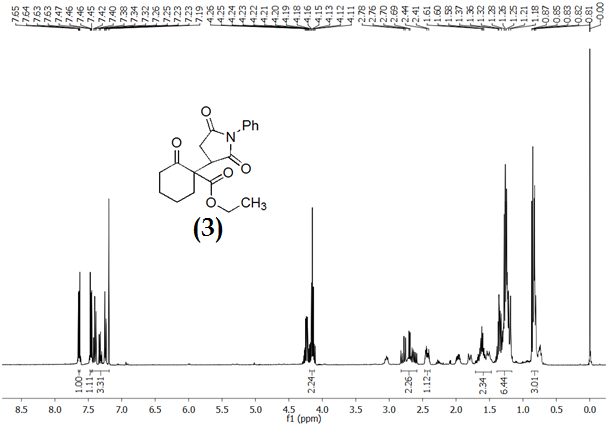


**^13^C NMR spectrum of compound 3**


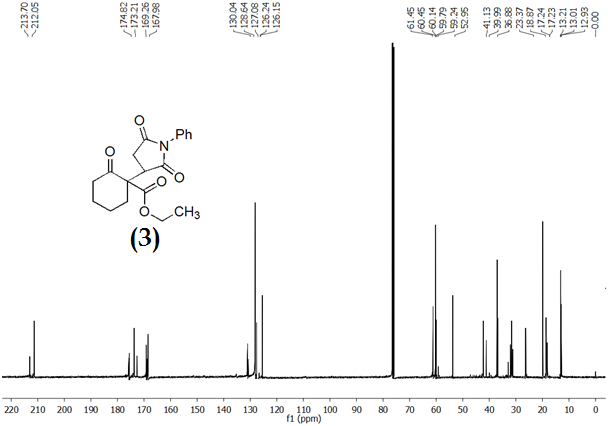


**^1^H NMR spectrum of compound 4**


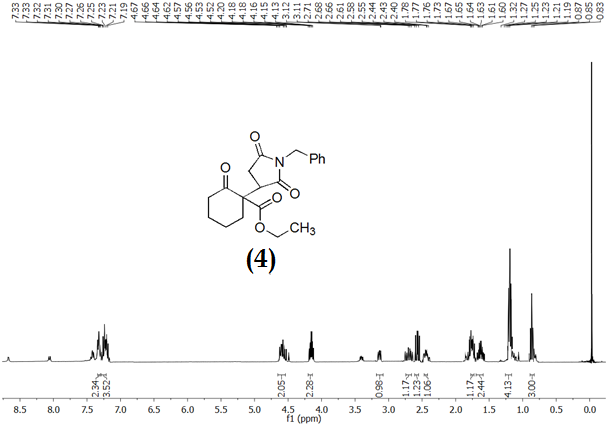


**^13^C NMR spectrum of compound 4**


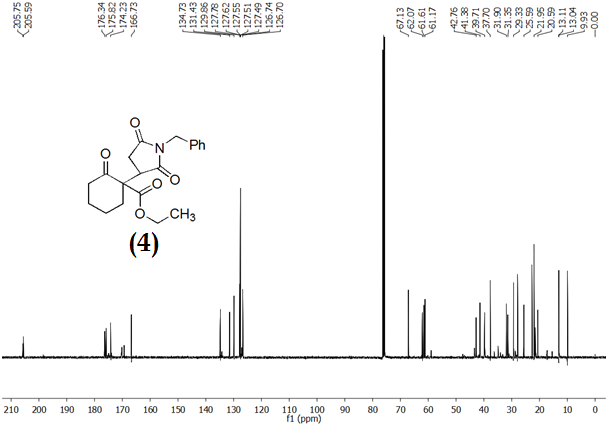

Supplement: Supplementary file 1 [file DataSheet1.docx]
